# The Role of Patch Testing in Evaluating Delayed Hypersensitivity Reactions to Medications

**DOI:** 10.1007/s12016-022-08924-2

**Published:** 2022-02-03

**Authors:** Carina M. Woodruff, Nina Botto

**Affiliations:** University of California San Francisco, San Francisco, CA USA

**Keywords:** Patch testing, Delayed hypersensitivity reactions, Cutaneous adverse drug reactions

## Abstract

Confirming drug imputability is an important step in the management of cutaneous adverse drug reactions (CADR). Re-challenge is inconvenient and in many cases life threatening. We review the literature on ideal patch testing technique for specific CADRs. Testing should be performed approximately 3 months after the resolution of the eruption using standard patch testing techniques. Commercially available patch test preparations are available for a minority of drugs, so in most cases, testing should be performed with the drug at various recommended concentrations and in different vehicles. Testing to all known excipients, such as dyes, vehicles and preservatives is also important. Immunosuppressive medications should be discontinued or down titrated to the lowest tolerable dose to decrease the risk of false negative reactions. We provide an overview of expert recommendations and extant evidence on the utility of patch testing for identifying the culprit drug in common CADRs and for specific drug or drug classes. Overall, there appears to be significant variability in the patch test positivity of different drugs, which is likely the result of factors intrinsic to the drug such as dermal absorption (as a function of lipophilicity and molecular size) and whether the drug itself or a downstream metabolite is implicated in the immune reaction. Drugs with high patch test positivity rates include beta-lactam antibiotics, aromatic anticonvulsants, phenytoin, and corticosteroids, among others. Patch testing positivity varies both as a function of the drug and type of CADR. The sum of the evidence suggests that patch testing in the setting of morbilliform eruptions, fixed drug eruption, acute generalized exanthematous pustulosis, and possibly also drug-induced hypersensitivity syndrome, photoallergic and eczematous reactions may be worthwhile, although utility of testing may vary on the specific drug in question for the eruption. It appears to be of limited utility and is not recommended in the setting of other complex CADR, such as SJS/TEN and leukocytoclastic vasculitis.

## Introduction

Cutaneous adverse drug reactions (CADRs) are a common clinical problem. Appropriately identifying the culprit drug is essential, yet this process can be fraught with error and difficulty due to the high frequency of polypharmacy and the lack of reliable diagnostic tests to confirm drug imputability. While oral or parenteral re-challenge is the gold standard, it is often inconvenient, unethical, and in some cases, life threatening. Patch testing has emerged as a viable and variably efficacious alternative. Herein, we provide an overview of the immunopathogenesis and key methodological issues underlying patch testing for drug reactions. We then discuss the variable utility and limitations of patch testing in the diagnosis of different types of cutaneous drug eruptions and for different classes of medications.

## Body

### Immunopathogenesis of Cutaneous Drug Eruptions

Immunologically mediated drug eruptions can be broadly classified into four categories based on the well-established Gell and Coombs criteria.[1] Type I reactions are mediated by Immunoglobulin E and histamine, and typically present with immediate-onset urticaria, angioedema or anaphylaxis. Skin-prick and intradermal testing are the studies of choice in establishing drug imputability in this setting. Type II reactions, such as drug-induced hemolytic anemia, are mediated by antibody-dependent cytotoxicity. Serum sickness is a classic example of a type III reaction, which results from complement activation by circulating antigen–antibody complexes. Patch testing is rarely helpful in the diagnosis of type I, II, or III reactions [2, 3].

Type IV reactions are delayed hypersensitivity responses mediated by T lymphocytes. Because they require the activation and expansion of antigen-specific T cells, these reactions typically occur days to weeks after exposure to the culprit drug or allergen. In addition to allergic contact dermatitis (for which patch testing is a well-established diagnostic tool), several adverse drug reactions with protean cutaneous manifestations fall into this category. This includes morbilliform (maculopapular) exanthems, fixed drug eruptions (FDE), acute generalized exanthematous pustulosis (AGEP), symmetric drug-related intertriginous and flexural exanthem (SDRIFE), Stevens Johnson syndrome/toxic epidermal necrolysis (SJS/TEN), as well as drug-induced hypersensitivity syndrome (DiHS, also formerly known as drug reaction with eosinophilia and systemic symptoms, or DRESS). Type IV reactions are mechanistically heterogeneous and further subdivided based on the unique cytokine milieu and effector cell populations by which they are characterized (Table 1) [4–8]. In reality, there is usually overlap between these subtypes, although one effector cell/cytokine milieu may predominate. Patch testing has shown to be variably efficacious in confirming drug imputability in the setting of these delayed reactions.

T lymphocytes can become sensitized to various components of the drug in question, including the active drug itself, excipients such as preservatives, dyes and other additives, as well as a drug metabolite. This sensitization is thought to occur via one of three unique mechanisms: 1) the drug itself can act as a hapten, directly binding to cell-bound or soluble cellular proteins and forming a hapten-carrier complex that can in turn bind to major histocompatibility complexes (MHC) on antigen presenting cells, leading to T cell activation, 2) a downstream metabolite of a chemically inert drug can act as a hapten as detailed in (1), or 3) rarely, certain drugs, by virtue of their structural features (rather than immunogenic capacity), can directly bind to certain T cell receptors or possibly MHC (4). Examples of the latter include large molecules such as mouse or rat derived antibodies.

**Table 1:** Subtypes of delayed hypersensitivity (type IV) reactions

Type IV subtype	Effector cells and cytokine milieu	Examples
Type IVa	T helper 1 (Th1) cells recruit monocytes by secreting large quantities of interferon-gamma (IFN-gamma), tumor necrosis factor-alpha (TNF-alpha), and interleukin-18 (IL-18)	No examples of pure type Iva-drug reactions Contact dermatitis (also type IVc)
Type IVb	T helper 2 (Th2) cells recruit eosinophils and mast cells via secretion of IL-4, IL-5 and IL-13 [5]	Morbilliform eruptions DiHS/DRESS
Type IVc	Cytotoxic CD4 + or CD8 + T cells induce keratinocyte apoptosis via granzyme B, perforin or Fas ligand, and granulysin [6, 7]	Contact dermatitis Fixed drug eruption SJS/TEN (may be a part of all type IV reactions)
Type IVd	T helper 17 (Th17) cells recruit neutrophils via IL-8 and granulocyte monocyte colony-stimulating factor (GM-CSF) release [8]	AGEP

### Patch Testing Technique for Delayed CADRs

**Fig. 1:**
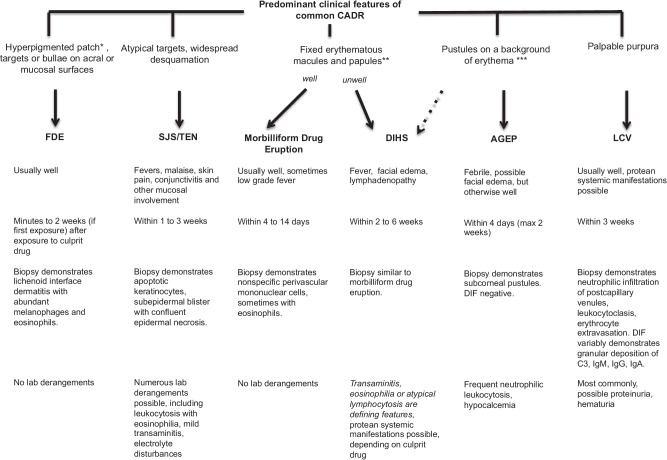
Predominant clinical features of common CADR

The first step in the determination of drug imputability is appropriate characterization of the eruption as a type IV delayed hypersensitivity reaction. One should consider the morphology and histopathology of the eruption as well as the presence of any associated laboratory derangements (see Fig. 1 and section on utility of patch testing for specific types of reactions below).

Key methodological considerations in the process of patch testing for delayed hypersensitivity reactions to medications include the timing of testing relative to the eruption, location of testing, allergen preparation and timing of patch test reads. Guidelines have been published by both the European Society of Contact Dermatitis (ESCD) [9] and the European Network on Drug Allergy (ENDA) [10] and are presented in Table 2.

**Table 2:** General guidelines for patch testing for delayed hypersensitivity reactions to medications

	European Society of Contact Dermatitis (2001) [9]	European Network on Drug Allergy (2002) [10]
Timing for testing	6 weeks to 6 months	3 weeks to 3 months
Concentration and vehicle for testing commercial form of drug	Pills: Coating should be removed and contents ground to a fine powder and tested:	Pills, capsules, liquid oral:
	- as is (undiluted, no vehicle)	- 0.5–30%, in petrolatum or water, depending on specific drug and formulation as described in literature
	- 30% in white petrolatum	
	- 30% in water	Parenteral:
		- Dilute in 0.9% NaCl
	Capsules: gel jacket should be moistened and tested as is. Powder contained inside should be tested:	- If non-hydrosoluble, dilute in dimethyl-sulfoxide (DMSO), then 0.9% NaCl
	- As is	
	- Diluted 30% in white petrolatum	
	- Diluted 30% in water	
	Liquid oral:	
	- As is	
	- Diluted 30% in water	
	Parenteral IV/IM/SC:	
	- 30% in water	
Concentration and vehicle for testing pure form of drug	10% in petrolatum, water + / − alcohol (depending on drug)	-
Timing for reads	Read at 20 min. If negative, then proceed with delayed readings on day 2, day 4 + / − day 7 (if negative on day 4)	Day 2 and day 3, occasionally day 4. Immediate reactions should be thoroughly ruled out by history

In general, it is recommended that testing be performed around 3 months following resolution of the eruption, depending on patient recovery and the severity of cutaneous manifestations (recommendations in the literature range from 3 weeks to 6 months). This timing is largely based on expert consensus; studies have not formally evaluated how or if patch test reactivity varies in the weeks to months following resolution of an eruption. Patients should ideally also be off all oral immunosuppressive agents such as prednisone, cyclosporine, methotrexate, and mycophenolate mofetil for at least 1 month prior to testing so as to minimize the risk of false negative results. In practice, the specific timing should vary relative to the half-life of the drug in question. The extant literature on the effect of specific immunomodulatory agents, including biologics, on patch testing is limited. As such, clinical practice is largely informed by guidelines developed on the basis of expert consensus [11]. While successful patch testing has been reported in the setting of low doses (10mg) of prednisone [12], evidence from a handful of studies suggests there is incremental dose-dependent inhibition and in some cases, complete suppression of allergic reactions to haptens with doses as low as 20 mg [13–15]. We recommend testing on the lowest possible dose of prednisone, ideally 10 mg or less. Observed attenuation or complete suppression of reactions has also been noted following topical application or intradermal or intramuscular injection of mid to high potency steroids for as few as 3 days prior to testing [16, 17]. Expert consensus recommendations are to avoid topical application of steroids for 1 week and wait 4 weeks after intramuscular triamcinolone or oral prednisone before pursuing patch testing [11].

Antihistamines do not affect patch test results and are safe to continue during testing. Recent UV exposure (including phototherapy) and/or sunburn or topical steroid use can likewise impair results and should be held for at least one to two weeks [11]. Additional contraindications include active infection, fever, or pregnancy (relative). While generally well tolerated, risks include recrudescence of the eruption, anaphylaxis (if the reaction was indeed a type I reaction and misclassified), and sensitization.

Patch tests are applied to the upper back and left in place for 48 h. One notable exception is in the case of FDE (and possibly SDRIFE), where it is recommended that patches be placed both on the upper back and on the previously affected skin site [18–20]. If possible, the European Society of Contact Dermatitis recommends additionally testing on the most affected site (of any eruption) to maximize yield [9, 21, 22].

Patch tests are prepared using Finn Chambers affixed with Scanpor tape (Epitest, Tuusula, Finland) or IQ Chambers (Chemotechnique), according to the methods used in the diagnosis of allergic contact dermatitis. Patches are left in place for a total of 48 h, and final reads are typically performed at 72 to 96 h, with an additional read at 168 (day 7) if prior reads are negative due to possibility of delayed positive reactions. The ESCD additionally recommends an early read at 20 m in the event of a possible immediate reaction, especially for tests with beta-lactam antibiotics [9]. Tests are typically interpreted via previously published internationally accepted criteria [17, 18]. In particular, palpable erythema and/or erythematous papules that cover less than 50% of the patch test surface are scored as a weak or 1 + reaction. Palpable erythema and/or erythematous papulovesicles that cover more than 50% of the patch test surface are scored as a moderate or 2 + reaction. Intense infiltration and/or a vesiculobullous reaction that involves the entire patch test or extends beyond it is scored as an extreme or 3 + reaction. Reactions presenting with only macular erythema are graded as questionable and those with macular erythema, accentuation of skin lines and/or glazed texture are typically irritant in nature.

### Drug-Specific Considerations

Commercially available patch test preparations are available for a minority of drugs. For example, Chemotechnique Diagnostics features a cutaneous adverse drug reaction series with 33 commonly implicated drugs, all diluted in a petrolatum base. Select drugs are also available from SmartPractice (Table 2). In the absence of commercially available tests, local pharmacies can assist in the preparation of precisely diluted haptens. In reality, investigators are often required to prepare patches from commercially available preparations of the drugs in question. Reassuringly, a recent study suggests these extemporaneous patch tests are diagnostically helpful and generally reliable [19].

Recommended concentrations are available in the literature for some drugs, in particular antibiotics [2, 10, 20–24] (Table 3).The ESCD and ENDA offer varying recommendations as a starting point for testing most other drugs, with concentrations ranging widely from 0.5 to 30.0% (Table 4). In the absence of specific recommendations, there is some consensus around testing the active ingredient in a 10% dilution and the commercialized drug (excipients included) in a 30% dilution [9]. In the setting of a severe cutaneous adverse reaction (SCAR), such as DRESS or SJS/TEN, starting with lower concentrations such as 0.1% and uptitrating to as high as 10% and/or performing open testing (whereby a small amount of the test substance is applied to intact skin and allowed to dry, rather than applied via Finn Chamber) is recommended in order to prevent recrudescence of the eruption [9, 10]. In reality, testing in the case of SCAR reactions like SJS/TEN is not recommended in clinical practice. Patch-testing induced relapses of prior eruptions have been reported more frequently with certain drugs—in particular, acyclovir, carbamazepine, pristinamycin and pseudoephedrine—so testing with lower concentrations is also recommended in these scenarios [20, 25–27]. Additional studies are needed to define optimal concentration and vehicles to ensure appropriate sensitivity and specificity of reactions. Overall, extant data seems to suggest that variations in the concentration of the active drug are probably not a key factor in false negative or positive results [19, 28].

**Table 3:** Commercially available patch test preparations for drugs implicated in cutaneous adverse drug reactions. Commercially available haptens are regularly updated. The above list reflects available allergens as of April 2021

Chemotechnique Diagnostics ©	SmartPractice ©
*Cutaneous adverse drug reaction series*	*Medicinal substances*
Amoxicillin trihydrate, 10.0% pet	Acetylsalicylic acid, 10% pet
dicloxacillin sodium salt hydrate, 10% pet	Bufexamac, 5% pet
Cefotaxim sodium salt, 10% pet	Dexpanthenol, 5% pet
Doxycycline monohydrate, 10% pet	Diclofenac, 2.5% pet
Erythromycin base, 10% pet	Etofenamate, 2% pet
Spiramycin base, 10% pet	Ibuprofen, 5% pet
Clarithromycin, 10% pet	Indomethacin, 2.5% pet
Pristinamycin, 10% pet	Naproxen, 5% pet
Cotrimoxazole, 10% pet	Paracetamol, 10% pet
Norfloxacin, 10% pet	Phenacetine, 10% pet
Ciprofloxacin hydrochloride, 10% pet	Phenylbutazone, 10% pet
Carbamazepine, 1% pet	Piroxicam, 1% pet
Hydantoin, 10% pet	Propanolol-hcl, 2% pet
Diltiazem hydrochloride, 10% pet	Propyphenazone, 1% pet
Captopril, 5% pet	Thymol, 1% pet
Acetylsalicylic acid, 10% pet	
Diclofenac sodium salt, 1% pet	*Antibiotics/antimycotics*
Ketoprofen, 1% pet	
Piroxicam, 1% pet	Ampicillin, 5% pet
Acetaminophen, 10% pet	Bacitracin, 20% pet
Acyclovir, 10% pet	Chloramphenicol, 5% pet
Hydroxyzine hydrochloride, 1% pet	Chlorotetracycline-hcl, 1% pet
Hydrochlorothiazide, 10% pet	Chlorquinaldol, 5% pet
Clindamycin phosphate, 10% pet	Clioquinol, 5% pet
Cefradine, 10% pet	Clotrimazole, 1% pet
Cefalexin, 10% pet	Erythromycin, 2% pet
Ibuprofen, 10% pet	Framycetin sulphate, 10% pet
Lamotrigine, 10% pet	Fusidic acid, 2% pet
Cefuroxime sodium, 10% pet	Gentamicin sulphate, 20% pet
Cefixime trihydrate, 10% pet	Kanamycin sulphate, 20% pet
Cefpodoxime proxetil, 10% pet	Metronidazole, 1% pet
Potassium clavunalate, 10% pet	Neomycin sulfate, 20% pet
	Nitrofurazone, 1% pet
	Nystatin, 2% pet
	Oxytetracycline, 2% pet
	Polymyxin-b-sulphate, 3% pet
	Streptomycin sulphate, 5% pet
	Sulfanilamide, 5% pet
	Tetracycline-HCl, 2% pet
	Tobramycin, 20% pet

**Table 4:** Recommended concentration and vehicles for drugs commonly implicated in cutaneous adverse drug reactions (de Groot) [25]

Drug category	Recommended concentration and vehicles in de Groot (25)
Antibiotics	Penicillin G—pure, 1% pet, 10,000iU pet
	Other penicillins—pure, 1% pet
	Cephalosporins—20% pet or pure, 0.5% water
	Cotrimoxazole—5% pet
	Tetracycline-HCl—3% pet, 5% pet
	Gentamycin sulfate—20% pet
	Erythromycin—1% pet, 5% pet, 10% pet
Other antimicrobials	Vancomycin—crushed 500 mg 15 and 30% pet
	Clindmycin HCL—1% water 1% pet
NSAIDs	Ibuprofen—5–10% pet
	Naproxen—2 and 5% pet
	Diclofenec—1–5% pet
Anticovulsants	Carbamazepine—1% pet
	Phenytoin—5 and 10% pet
Benzodiazepines	Tetrazepam—0.5%, 1%, 5%, 10%, 20% pet and pure
	Diltiazem—1% pet
Other	Hydroxyzine—2/5% pet
	Pseudoephedrine—crushed 60 mg tab, 30% pet
	Heparin derivatives—commercial prep pure
	Captopril—10% water, 5% pet, 10% pet

Dermal absorption is required to elicit a reaction, but in many cases, the optimal vehicle to enable penetration has not been defined. Thus, in most cases, it is advisable to test with multiple vehicles (aqueous and petrolatum or white soft paraffin). Penetration crucially depends on the lipophilicity and the molecular weight of a drug. A process called tape stripping, whereby cellophane tape is applied to the skin and removed 10 to 15 times prior to patch placement, is recommended to augment the penetration of water soluble or very large drugs (i.e., those with a molecular weight greater than 500 Da) [20].

In general, pills should have their coating removed and the contents should be ground to a fine powder and tested as is, in addition to being tested within aqueous and petrolatum bases. For capsules, both fragments of the gel jacket (moistened) and the capsule contents should be tested separately (in a manner similar to that of pill contents). Liquid preparations should be tested at full concentration and in an aqueous dilution at varying concentrations. If available, the ENDA recommends additionally testing with the parenteral formulation of a drug (even if not taken by the patient) due to superior standardization [10]. Testing should be performed within 1 day of patch preparation due to unknown stability of drug components.

Whenever possible, an effort should be made to obtain samples of the pure drug as well as excipients, such as dyes, vehicles, and preservatives. The ESCD recommends testing active drugs in lower concentrations and in various vehicles: 10% petrolatum, 10% aqueous, and in some cases, 10% alcohol dilutions. The latter has been shown to increase diagnostic yield for certain medications; in particular, steroid hormones such as estrogens and progesterone [29]. It may be helpful to test other drugs within the same pharmacological family to assess for cross-reactivity [9].

### Utility of Patch Testing for Specific Drugs

The utility of patch testing for cutaneous adverse drug reactions in part depends on the drug being tested. Factors intrinsic to the drug such as lipophilicity, molecular weight and whether the drug itself or a downstream metabolite is implicated in the immune reaction (which the skin may not be able to generate in the same manner that systemic ingestion does to elicit a T cell response) likely account for this. One notable example is allopurinol, which even in the setting of high imputability typically is not reactive on patch testing because the immunogenic form of the drug is the metabolite oxypurinol, which is only generated in the liver [30–32].

Among the antibiotics, rates of positivity seem to be particularly high for beta-lactams (cephalosporins and amoxicillin in particular), pristinamycin and clindamycin [3, 25, 33, 34–40]. Some studies suggest sulfonamides and macrolides (with exception of spiramycin) may be less likely to yield positive results, possibly due to metabolites being the immunogenic culprit [34, 41, 42].

Other drugs with high patch positivity rates include the aromatic anticonvulsants (carbamazepine [35, 41, 43–45] and phenytoin [46–48]), corticosteroids [49, 50], tetrazepam [21, 25, 51, 52], diltiazem [41, 53], captopril [41], hydroxyzine [54], pseudophedrine [55, 41], and heparin derivatives [56, 57].

Importantly, rates of patch test positivity for each drug also vary depending on the type of reaction in which the drug is implicated. For example, high rates of patch test positivity have been reported for carbamazepine in the setting of DRESS, but not consistently for SJS/TEN [58].

### Utility of patch Testing for Specific Types of Reactions

Appropriately characterizing a drug eruption is essential for timely diagnosis and management and will also aid in determining whether confirmatory patch testing is useful once the eruption has resolved. In approaching these cases, we recommend first categorizing the eruption on the basis of clinical morphology and histopathologic findings (Fig. 1). It is essential to inquire about all prescription and over the counter medications to which the patient may have been exposed, including suppositories, drug-releasing implants, injections, patches, eye drops, otic preparations and recreational drugs. Unlike drug-induced urticaria or anaphylaxis, which typically occur minutes to hours after drug exposure, there can be a weeks’ long temporal delay between exposure to the inciting drug and development of the eruption. Creating a drug chart with the timing and duration of all drug exposures can be extremely helpful in narrowing down potential culprit medications. Regularly updated online databases with reported reaction types for particular drugs such as Litt’s Drug Eruption and Reaction Database are extremely helpful in further narrowing down imputability.

#### Simple CADRs

Simple CADRs are those that present with only cutaneous involvement.

1. Morbilliform/maculopapular drug eruptions
  Morbilliform (or maculopapular) exanthems are characterized by numerous pruritic erythematous papules and plaques in the absence of facial edema, mucosal involvement, systemic symptoms, or laboratory derangements (in contrast to DIHS/DRESS). Involvement typically begins on the trunk or proximal extremities and generalizes over the course of 48 h. It is typical for these exanthems to emerge within the first 2 weeks of exposure to the culprit drug. Rarely, onset can occur up to 10 days after drug discontinuation. Well-established culprit drugs include aminopenicillins, cephalosporin, sulfonamides, anticonvulsants, and allopurinol.
  The role of patch testing in the diagnosis of these eruptions has been relatively well studied in several case–control studies. Reported rates of patch test positivity in these studies range from 10 to 59%; it is unclear if this high degree of variability is the result of differences in patch technique and or study design, or more likely, differences in the drugs that were tested, since patch test positivity of specific drugs seems to vary widely and depend on various factors, including dermal absorption of the drug, whether the drug or a metabolite is the immunogenic trigger, etc. [26, 41, 59, 60]. For example, a high rate of patch test positivity seems to be consistently reported for studies evaluating the imputability of antibiotics in particular—e.g., all antibiotics (21.8 to 46.2%) [34, 61], aminopenicillins (34.7–55.0%) [10, 37], beta-lactams (31.2 to 100%) [62, 63], clindamycin (66.7%) [64], and pristinamycin (60.0%) [65]. In the broader studies, nonantimicrobial drugs with high patch test positivity rates include captopril, diltiazem, pseudoephedrine, and carbamazepine [41].
2. Fixed drug eruption (FDE)
  Fixed drug eruptions classically present with a recurrent erythematous patch or patches that evolve into targets and sometimes bulla at the same body site with each exposure to the offending drug. These lesions have a predilection for the oral and genital mucosa and tend to occur minutes to hours after drug exposure. Commonly implicated drugs include sulfonamides, NSAIDs, tetracylines, erythromycin, fluoroquinolone, cetirizine, hydroxyzine, and barbiturates.
  Patch testing appears to be fairly helpful and safe in the setting of FDE. In a study of 52 patients, 40.4% [21] were patch test positive, mostly to NSAIDs (nimesulide, piroxicam, and etoricoxib) and 1 to cetirizine [66]. In a study of 34 patients, 27 (79.4%) exhibited patch test positivity, mainly to NSAIDs (63% of all positives) and paracetamol (14.8%) [67]. In another study looking at sulfonamides in particular, 20% (5/25) of the patients who were patch tested were patch test positive. Based on results, the authors suggest testing may be more fruitful if there are residual lesions at the time of patch testing [68]. In a study of 30 patients with open patches on lesional skin, a positivity rate of 86.7% was reported, mostly to phenazone salicylate (16 of 16 tested) and carbamazepine (3 of 3 tested), and doxycycline (2 of 3 tested) [18]. In smaller studies, high rates of positivity to piroxicam (85.7% in one study, 6 of 7 patients) [69] and antibiotics (beta-lactams, fluoroquinolones and sulfonamides) (50%, corresponding to 4 of 8 patients) have also been reported [61]. There are numerous case reports of patch test positivity in the setting of FDE to other drugs, including fluconazole [70–72], adalimumab [73], etoricoxib [74–78], piroxicam [69, 79, 80], nimesulide [81–83], and contrast medium [84, 85].
  The importance of the test vehicle for certain drugs is highlighted in a few studies by Ozkaya-Bayazit et al. In one study of 27 patients with cotrimoxazole-induced FDE and 20 healthy controls, no positive results were obtained when testing with petrolatum, but 25 of 27 tested patients (92.6%) exhibited positivity when the drug was tested in varying concentrations in DMSO [86]. In a different study, all 4 of 4 patients patch tested to metamizole and 3 of 5 patch tested to naproxen in DMSO at a concentration of 20% were patch test positive, while none exhibited positivity when the causative drugs were tested in petrolatum [87]. However, in the aforementioned study by Alanko (1994), the vehicle was not important for most allergens, with the exception of carbamazepine, sulfadiazine, and trimethoprim, in which case more reactivity was seen with DMSO, which also produced irritant reactions in controls [18].
  Concentration likewise seems important; Mahboob et al. tested over 300 patients with clinically diagnosed FDE to different concentrations of drugs in white soft paraffin and found a concentration-dependent effect on patch test positivity, with less than 1% patch test positivity rate with 1% concentration versus 59.7% positivity rate for 5% concentration [88].
3. Photosensitivity
  Medications can induce photosensitivity, as well as photoallergic and non-immunologic, dose-dependent phototoxic reactions. Patch testing appears to be helpful in the diagnosis of drug-induced photosensitivity and photoallergic reactions, both of which are typically mediated by UVA rays. The most commonly implicated drugs include NSAIDs, TMP-SMX, tetracyclines, phenothiazines, quinine, quinidine, and some sulfonylureas.
  In the investigation of these reactions, it is recommended that patients be tested both to the drug itself (as a control) and separately to the drug, followed by irradiation on day 1 or day 2 of testing with 5 to 10 J/cm^2^ of UVA. In Barbaud et al. (1998), 4 of the 4 (100%) patients suspected of having drug-induced photosensitivity exhibited positive photopatch tests (1 to oestradiol, methoxsalen each, and 2 to hydroquinidine) [26]. In a study evaluating antibiotics in particular, 2 of 7 patients with suspected photosensitive eruptions were patch test positive [34]. Broader studies of photoallergens (including but not restricted to drugs) suggest such high rates of positivity can also be seen with phenothiazines [89] and NSAIDs [90].
4. Lichenoid drug reactions
  Various drugs have also been implicated in lichenoid drug reactions, which typically present with generalized or photoaccentuated violaceous papules and plaques with Wickhma’s striae and variable mucosal involvement. Common culprits include gold, hydrochlorothiazide, furosemide, NSAIDs, aspirin, ACE inhibitors, calcium channel blockers, beta-blockers, terazosin, quinidine, proton pump inhibitors, pravastatin, phenothiazines, anticonvulsants, antituberculous drugs, and antimalarials. The literature on the utility of patch testing in this setting is sparse. There are scattered reports of patch test positivity to various implicated drugs, including carbamazepine [91], misoprostol [92], tiopronin [93], and aminoglycoside antibiotics [94].
5. Eczematous eruptions
  The utility of patch testing topical drugs in the diagnosis of eczematous eruptions is well-established. Both the active drug and excipients can be implicated. Examples include topical corticosteroids, antimicrobials such as antibiotics and antifungal agents, as well as anesthetics. The North American Contact Dermatitis Group standard series screens for several of the more common topical drug allergens; supplemental testing with dedicated topical medicament and corticosteroid series is available via Dormer and Chemotechnique. We recommend testing the patient’s topical medicament as is, in addition to the active drug and any excipients in its formulation.

Oral drugs have also been implicated in eczematous eruptions, with a few studies and scattered case reports suggest patch testing can be helpful in uncovering the culprit drug. For example, Barbaud et al. (1998) report that 3 of 9 patients with eczematous reactions had positive patch tests to triamcinolone, enoxaparin and penicillin G [26]. In another study, 3 of 3 patients with eczematous eruptions to pristinamycin were positive on testing [65]. In another study of antibiotics, Grandhe et al. found that 33% (2 of 6) patients were patch test positive [61]. Positivity to various other drugs, including isoniazid and ethambutol [95], dipyridamole [96], and carbamazepine [97] have been reported.

However, it is important to note that patch testing does not reliably identify all culprit drugs implicated in eczematous eruptions. For example, patch testing does not appear to be helpful in confirming imputability of hydrochlorothiazide and calcium channel blockers, both of which are arguably more frequently implicated in chronic eczematous dermatoses [98].

#### Complex CADRs

Complex CADRs are a potentially life threatening category of eruptions that typically present with a mixture of cutaneous and visceral/systemic involvement.

1. Drug induced hypersensitivity syndrome (DIHS/DRESS)
  The cutaneous morphology of DIHS can mimic a morbilliform drug eruption, but is importantly accompanied by facial edema, fever, and evidence of end-organ involvement, including most commonly, eosinophilia (> 1500 absolute eosinophils), lymphocyte activation (manifested as atypical lymphocytosis, lymphadenopathy, and/or lymphocytosis) and hepatitis. In terms of other skin findings, follicular accentuation, superficial desquamation, or superficial pustules can sometimes also be seen. Other acute forms of end-organ involvement can include interstitial nephritis, colitis, encephalitis, interstitial pneumonitis, myocarditis, and silaladenitis, often varying depending on the culprit drug in classic patterns. DIHS tends to start 2 to 6 weeks after exposure to the inciting drug and can last for many months. Reported late sequelae include thyroiditis, diabetes mellitus and syndrome of inappropriate secretion of antidiuretic hormone (SIADH). Frequently implicated drugs include sulfonamides, certain anticonvulsants, allopurinol, abacavir, nevirapine, dapsone, azathioprine, and minocycline.
  Several large prospective studies have evaluated the utility of patch testing in the diagnosis of DIHS, suggesting it can be fruitful. In the largest multicenter study, 64% of patients (46 of 72) were found to be patch test positive, with the majority of reactions to beta-lactams [14], carbamazepine [11], proton pump inhibitors [5], vancomycin [4], and pristinamycin [3] [58]. Other studies confirm the utility of testing for DIHS related to antimicrobials and carbamazepine in particular. In a study of 56 patients with mostly anticonvulsant induced DIHS, a patch test positivity of 31.1% (18 of 56) was reported, with 13 reactions to carbamazepine, 2 were to lamotrigine, and 1 to phenytoin and topiramate each [99]. In a study of antibiotic-induced DIHS, a positivity rate of 31.6% (6 of 19) was reported, with positives to amoxicillin [4], cotrimoxazole [1], ceftriaxone [1], and ciprofloxacin [1] [34]. Despite high imputability, patch testing with allopurinol appears to be low yield, likely due to a drug metabolite being implicated in the eruption [58, 99, 100].
2. Acute generalized exanthematous pustulosis (AGEP)
  Patients with AGEP typically present with innumerable and sometimes subtle non-follicularly based subcorneal pustules on a background of erythema. Facial edema, fever, neutrophilia and/or eosinophilia, and hypocalcemia frequently accompany these cutaneous findings. In contrast to DIHS, the eruption is self-limited and typically develops within 2 days to 2 weeks of exposure to the inciting drug. Ampicillin/amoxicillin, diltiazem, sulfonamide antibiotics, terbinafine, imatinib, hydroxychloroquine, quinolones, and pristinamycin are among the most commonly implicated drugs.
  A few small studies suggest patch testing is generally high yield in this setting. In a study of 14 patients with AGEP, 50% were positive on patch testing to the following drugs: amoxicillin (2), spiramycin (1), virginiamycin (1), carbamazepine (1), phenobarbital (1), diltiazem (1) [101]. In a study of 16 patients patch tested by commercially or pharmacy prepared patch tests as well as extemporaneous tests with variable dilutions prepared by a nurse, the positivity rate for patients with AGEP was 43.8%, with positives to amoxicillin, pristinamycin, spiramycin, and hydroxyzine [25]. The largest study to date evaluated 45 patients and reported a positivity rate of 58%, with positives to pristinamycin (8), corticosteroids (3), radiocontrast media (2), acetaminophen (2), fluindione (1), nonfractionated heparin (1), pseudoephedrine (1), tetrazepam (1), clindamycin (1), and varenicline (1) [58]. In the same study, 5 additional cases were diagnosed with delayed intradermal tests, suggesting this may be a helpful adjunctive study in the setting of negative patch tests [58]. In some of these studies, the patch test positivity rate for patients with AGEP was significantly higher than for those with other complex drug eruptions [25, 101]. In contrast, in a study specifically looking at antibiotics, the positivity rate for AGEP was 18.2% (2 of 11 reactions; ciprofloxacin and dicloxacillin) and lower than for other drug eruptions [34].
  In addition, the literature is replete with case reports of patch test positivity to various agents in the setting of AGEP, including terbinafine [102, 103], contrast media [104, 105], hydroxychloroquine [106, 107], acyclovir [108, 109], metronidazole [110, 111], diltiazem [110, 112], cephalosporins [113–115], amoxicillin-clavulanate [116, 117], and metamizole [87, 118].
3. Stevens–Johnson syndrome/toxic epidermal necrolysis (SJS/TEN)
  SJS and TEN are life-threatening hypersensitivity reactions characterized by fever, atypical macular or papular targets, and painful cutaneous desquamation and mucosal erosions. These syndromes typically develop 1 to 3 weeks after exposure to the inciting drug. Given that SJS and TEN are frequently caused by the same medications and have indistinguishable histopathologic findings, they are best thought of as closely related entities occurring along a spectrum of severity (SJS affecting less than 10% body surface area, while TEN affects more than 30%). Common culprits include TMP-SMX, penicillins, sulfonamide antibiotics, carbamazepine, lamotrigine, nevirapine, non steroidal anti-inflammatories, and allopurinol.
  The literature on patch testing for SJS/TEN is sparse. A prospective, multicenter case–control study including 17 patients with SJS/TEN reported a positivity rate of 23.5%[58]. Another prospective case–control study focusing particularly on aromatic anti-epileptic agents reported a patch test positivity of 62.5% (10/16) for carbamazepine in particular [100]. In a study of 22 patients with SJS, only 2, corresponding to 9%, had positive patch test results to sulfonamide and phenobarbital [101]. In a study particularly focusing on antibiotics, 20% (1 of 5) patients with SJS was exhibited positivity, in this case to meropenem [34]. In general, given sparse data, variable reported yield and the potentially disastrous consequences of re-eliciting the eruption with testing, we do not recommend patch testing in this setting.
4. Drug-induced leukocytoclastic vasculitis (LCV)
  LCV classically presents with palpable purpura. Drugs are among several known triggers. Drug-induced LCV typically occurs within 3 weeks of exposure to the inciting drug. Fevers, arthralgias, peripheral neuropathy, colitis and nephritis may be accompanying features. While there are scattered case reports of patch test positivity to various drugs implicated in LCV, including propylthiouracil [119], topical NSAIDs [120] and others [121], given the dearth of evidence regarding optimal methodology and safety of patch testing in this setting, we do not recommend it as a tool to investigate drug imputability for vasculitis.

### Safety of Patch Testing for CADRs

Although rare, there are several reports of patch testing inducing recrudescence of the original cutaneous adverse reaction. In a study of 72 patients with various cutaneous adverse drug reactions, 5 patients experienced a mild relapse of their eruption with patch testing (2 had a history of morbilliform eruption, to acyclovir and pseudoephedrine, respectively; 2 had a history of eczematous eruption, to amoxicillin and triamcinolone, respesctively; and 1 with an erythrodermic eruption to hydroxyzine) [26]. In this study, all above drugs were tested in 30% concentration in either petrolatum or aqueous medium. There are several reports for other drugs where patch testing induced recurrence of the original cutaneous reactions, including carbamazepine implicated in an exfoliative dermatitis [35], acetaminophen-induced AGEP [122], metamizole in FDE [123], ranitidine-induced DIHS [124], pseudoephedrine implicated in SDRIFE [125], and pristinamycin-induced AGEP requiring systemic corticosteroids [126].

Patient factors may also be an important consideration. In a study of patients patch tested for suspected complex cutaneous reactions to antituberculosis drugs including DIHS, and SJS/TEN, in 10 of the 11 HIV-positive patients that were tested, systemic reactions, including rash, eosinophilia, transaminitis, and fever noted with patch testing [127].

In sum, in the setting of the above drugs, complex drug reactions and in certain populations, it may be prudent to test with lower drug concentrations or avoid patch testing altogether.

## Discussion

Expanding literature attests to the utility of patch testing in the confirmation of drug imputability in CADRs. Patch testing appears to be generally fruitful in the setting of morbilliform eruptions, FDE, AGEP, and possibly also DIHS, photoallergic and eczematous reactions. It appears to be of limited utility and is not recommended in the setting of SJS/TEN and leukocytoclastic vasculitis. In addition to reaction type, testing for imputability of certain drugs also appear to be more high yield than others—beta-lactams, anticonvulsants (in particular carbamazepine) and NSAIDs in general appear to be more likely to result in positive patch test reactions. Other drugs, such as allopurinol, consistently do not patch test positive, likely because of factors intrinsic to the drug, such as lipophilicity or immunogenicity requiring conversion to a metabolite.

In general, a positive test is very helpful in establishing causality. False positives have been reported for patch tests to certain drugs, including commercial formulations of various drugs containing irritants such as sodium lauryl sulfate, or colchicine and misoprostol [128, 129]. Testing with controls (such as patients who were exposed to a suspected culprit drug but did not experience a cutaneous adverse reaction), as was done in most of the aforementioned studies, is thus advisable [3], but often not feasible in a clinical setting.

In contrast, the negative predictive value of patch testing for drug reactions is low. False negative reactions can occur due to a multitude of reasons, including suboptimal testing technique (for example, low concentration, insufficient percutaneous absorption) or because the reaction itself is due to a drug metabolite, or secondary to other transient potentiating factors that cannot be replicated. For example, concomitant viral infections (such as EBV, HIV, CMV, HHV6) and flaring autoimmune conditions can potentiate delayed hypersensitivity reactions, possibly by promoting widespread T cell activation and increased expression of MHC and co-stimulatory molecules [130–132].

In the setting of a negative patch test, skin prick or intradermal testing can be pursued, although false positives are more common with intradermal testing than with patch testing. Details of skin prick and intradermal testing are beyond the scope of this review as they are generally more helpful for urticarial reactions. However, these modalities have demonstrated some efficacy in identifying the culprit drug in the setting of morbilliform eruptions and fixed drug eruption. For example, in one study, five of twelve patients (42%) with morbilliform eruptions who had negative patch test results subsequently exhibited positive skin prick or intradermal tests to the suspected drugs. In contrast, a much higher proportion, 11 of 13 (85%) of patients with drug-induced urticaria who were negative on patch testing were positive on skin prick and/or intradermal testing [26]. Given the high risk of rash recrudescence, these tests are contraindicated in the setting of SCARs, as well as erythema multiforme and leukocytoclastic vasculitis. Importantly, given the risk of anaphylaxis, intradermal tests should only be performed in settings where a crash cart is available.

In the future, various limitations need to be addressed in order to enable more widespread adoption of confirmatory testing. Currently, the variability in study design and lack of standardized patch testing methodology in the literature precludes direct comparison between studies. In addition, only a limited number of haptens are commercially available for testing. In many cases, pharmacies in academic medical centers or specialized compounding pharmacies in the community can be helpful in the preparation of haptens. However, both the availability of centers and cost of case-by-case compounding are limitations to more widespread use.

More work is also needed in order to define optimal practices in testing, such as the optimal concentration and vehicle for testing particular drugs. Additional studies are also needed to elucidate the role of metabolites versus the native drug in precipitating eruptions. Testing key metabolites may enable confirmation of testing in previously low yield scenarios. For example, Lee et al. (2003) uncovered 3 additional relevant reactions in a cohort of 13 patients with suspected cutaneous adverse reactions to carbamazepine by additionally testing to carbamazepine’s main metabolite, carbamazepine-epoxide [133]. Overall, given how rare these reactions are, collaborative, multi-centered studies will be required to achieve these goals.

## Summary and Conclusions

In summary, based on the extant literature, patch testing can be a helpful and generally safe diagnostic tool in the evaluation of CADRs. Testing can be considered on a case by case basis in the setting of morbilliform eruptions, FDE, AGEP, and possibly also DIHS, photoallergic and eczematous reactions. Given the risks of rash recrudescence and low yield of testing, we do not recommend patch testing for drug imputability in the setting of SJS/TEN and leukocytoclastic vasculitis. Evidence suggests that the technique should be tailored based on the clinical features of the eruption, drug culprits in question and certain patient features.
